# Cancer and the FRA3B/FHIT fragile locus: it's a HIT

**DOI:** 10.1038/sj.bjc.6600937

**Published:** 2003-05-13

**Authors:** K Huebner, C M Croce

**Affiliations:** 1 Kimmel Cancer Institute, Jefferson Medical College, Philadelphia, PA 19107, USA

**Keywords:** fragile sites, tumour suppressor, FHIT, carcinogenesis, epithelial cancers, HIT, histidine triad

## Abstract

The FHIT gene encompassing the most active common human chromosomal fragile region, FRA3B, was discovered in 1996 and proposed as a tumour suppressor gene for important human cancers. Seven years and more than 350 reports later, early questions concerning its tumour suppressor role have been answered. Recent studies on the role of Fhit loss in major types of human cancers report association with high proliferative and low apoptotic indices, node positivity, loss of mismatch repair protein, likelihood of progression and reduced survival.

Soon after discovery, it was shown that the FRA3B/FHIT locus frequently exhibits deletions in preneoplasias and cancers, Fhit protein expression is lost or reduced in the majority of human cancers, and the orthologous mouse Fhit locus is fragile and exquisitely sensitive to carcinogen damage (Huebner *et al*, 1998; Huebner and Croce, 2001).

In the last 3 years, more than 180 papers featuring Fhit have appeared, as have publications on possible cancer association of genes at other common fragile sites, and Fhit has been shown unequivocally to act as a tumour suppressor. Fhit knockout mice show increased susceptibility to spontaneous and induced tumours, and oral gene therapy using adeno and adenoassociated FHIT viruses prevents and reverses induced forestomach tumours in Fhit +/− mice (Fong *et al*, 2000; Dumon *et al*, 2001).

So what does this mean for diagnosis, prognosis and therapy of human cancers? Here we review the types of cancers in which Fhit loss is associated with specific clinical features, and consider the importance of further investigation of consequences of Fhit loss in these cancers (Figure 1

**Figure 1:**
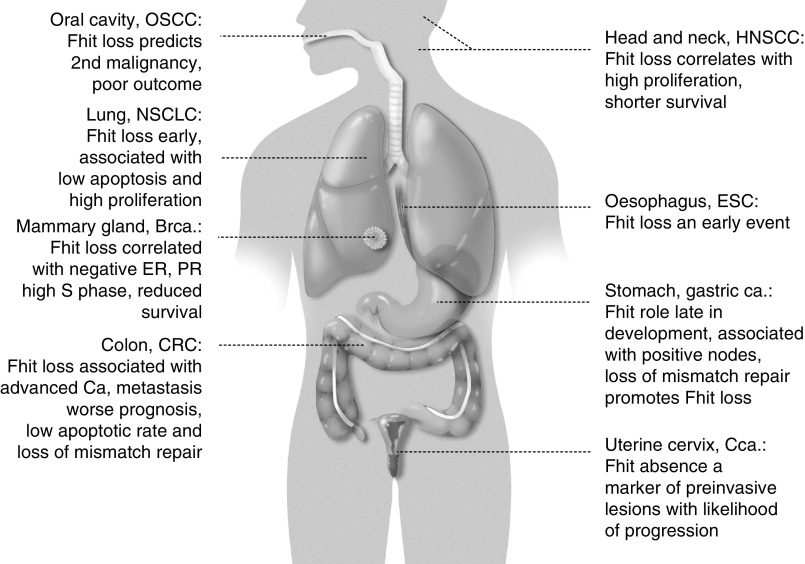
Human tissues with especially vulnerable FHIT genes.

).

## UPPER GASTROINTESTINAL TRACT

Oral, oesophageal and gastric cancers are common in some Asian countries and parts of Africa, and environmental agents, such as alcohol and tobacco use, as well as certain dietary habits, are suspected to play important roles.

In an early study using RNA extracted from oral carcinomas in South Africa, FHIT transcription was examined by reverse transcription of RNA and PCR amplification of the cDNA product (RT–PCR) using primers within the FHIT open reading frame. In a more recent study, these investigators (van Heerden *et al*, 2001) compared the frequency of detection of FHIT aberrant expression using the RT–PCR assay for mRNA aberration and analysis of level of Fhit protein expression by immunohistochemical analysis in tissue sections using several polyclonal anti-Fhit sera. The findings were that 71% of the oral squamous cell carcinomas (OSCCs) showed reduced or absent Fhit protein and half of the cases with reduced Fhit showed aberrant RT–PCR products. Thus, immunohistochemical analysis of Fhit expression is more sensitive for the detection of alterations in expression (see Table 1

**Table 1:** FHIT alterations in primary cancers: association with clinical features (2000–2003 update)

**Site/tissue**	**Alterations**	**Correlations/conclusions**	**References**
Oral cavity	LOH, aberrant RT–PCR in premalignant lesions, SCCs		Tanimoto *et al* (2000)
Tongue cancer	Fhit reduced/absent in 68%	Predicts poor outcome	Lee JI *et al* (2001)
Betel/ tobacco-associated	41% OSCCs reduced Fhit, aberrant RT-PCR in 50% preinvasive lesions		Chang *et al* (2002)
OSCC	3p, 9p LOH in post-treatment premalignant lesions	Predicts second oral malignancy	Rosin *et al* (2002)
	71% OSCCs reduced or absent Fhit		van Heerden *et al* (2001)
HNSCC	Fhit low in 53%	Correlated with high Ki67	Mineta *et al* (2003)
ESC	FHIT LOH in 76%, Fhit low in 70%, >loss in tobacco/alcohol users	Fhit loss an early event	Mori *et al* (2000)
	LOH or aberrant RT–PCR in 77%	Fhit a target of carcinogens	Menin *et al* (2000)
	Reduced/absent Fhit in 78%	Not correlated with prognosis, smoking history; associated with progression	Shimada *et al* (2000)
	Fhit loss in 89% invasive, 68% CIS, 43% dysplasias	Associated with prognosis; not with p53 expression, clinicopathological features or apoptosis	Kitamura *et al* (2001)
Lung/ head and neck	Most showed Fhit loss, low p21, increased cell proliferation	Low Fhit correlated with low apoptosis, high proliferation. HNSCC cases with low Fhit showed shorter survival	Pavelic *et al* (2001)
NSCLC	52% Fhit negative	Correlated with FHIT LOH, inversely with KRAS mutation; not with clinical parameters	Geradts *et al* (2000)
	FHIT allelic imbalance in 64%	Association with p53 overexpression	Garinis *et al* (2001)
	Fhit reduced in 67% of asbestos exposed, 59% in nonexposed	FHIT inactivation contributes to ca. development	Pylkkanen *et al* (2002)
Breast cancer	25% bilateral cases showed concordant FHIT LOH	Suggests role in bilateral breast cancers	Kollias *et al* (2000)
	FHIT LOH studied	FHIT LOH correlated with ER, PR negativity, high S-phase fraction, reduced survival; 60% increased risk of dying	Ingvarsson *et al* (2001)
	Fhit low in 67%, promoter hypermethylated in 48%	FHIT inactivated biallelically by LOH, hypermethylation	Yang *et al* (2002)
Brca1 deficient	Fhit low in ∼90% of cases	BRCA1 pathway important in protecting FHIT from damage	Turner *et al* (2002)
Gastric cancer	Fhit reduced in 63%	Alteration in FHIT can play an important role	Lee SH *et al* (2001)
	Fhit expressed in preneoplastic lesions	Suggests role in late carcinogenesis	Caselli *et al* (2001)
	FHIT LOH in 89%; Fhit low in 78%	Associated with positive node status; lack of mismatch repair could promote FHIT alterations	Huiping *et al* (2002)
Colon adenomas	Fhit low in 47%	Fhit inversely correlated with degree of dysplasia; altered Fhit occurs early in CRC development	Morikawa *et al* (2000)
CRC	Fhit reduced in 44% carcinomas, few ACF and adenomas; reduced in most metastatic lesions	Decreasing Fhit with decreasing differentiation and in tumours with metastasis; low Fhit associated with degree of dysplasia	Hao *et al* (2000)
	Aberrant RT–PCR in 52%; 46% FHIT genomic alteration	No correlation with clinicopathologic characteristics	Luceri *et al* (2000)
	50% positive for Fhit; Msh2 loss correlated with Fhit loss	Fhit loss correlated with progression; mismatch repair important in stability of FHIT	Mori *et al* (2001)
	Fhit loss in ∼18% well, moderately differentiated, 81% of poorly diff. ca. Loss of Mlh1 in 40%	Fhit loss associated with advanced ca. and absence of Mlh1; not with p53 expression	Andachi *et al* (2002)
	Fhit absence/reduction in poorly diff. ca.	Correlated with distant metastasis, worse prognosis. Directly proportional to apoptotic rates	Mady and Melhem (2002)
Cervical cancer	Fhit low in 71% invasive cancers, 52% HSILs associated with inv. ca.	Fhit loss more frequent in HSILs associated with progression to inv. ca.	Connolly *et al* (2000)
	Fhit reduced in 83%	FHIT alterations important in cervical ca.	Helland *et al* (2000)
	FHIT gene altered in 67%	FHIT inactivation involved in cervical ca.	Herzog *et al* (2001)
	Fhit low in 66% stage II–III ca.	Poor prognostic factor	Krivak *et al* (2001)
	FHIT LOH frequent, occurred in CIN synchronous with inv. ca.	Suggests essential role in clonal selection and early cervical ca.	Guo *et al* (2001)
	Aberrant RT–PCR in 20–30% CIN 2/3 lesions; Fhit loss rare	No association with HPV; could be an independent risk factor	Terry *et al* (2002)
	Fhit low in 50% CIN3, 78% MICA lesions; 87% cases with low Fhit positive for HPV16	Associated with HPV16 in CIN1, 2, 3 and MICA; FHIT a cofactor with HPV16	Butler *et al* (2002)

CIN=cervical intraepithelial neoplasia; HSIL=high-grade squamous intraepithelial lesion; MICA=microinvasive carcinoma; OSCC=oral squamous cell carcinoma; ESC=oesophageal squamous cell carcinoma; CRC=colorectal carcinoma; ACF=atypical crypt foci; NSCLC=nonsmall cell lung carcinoma; IPF=idiopathic pulmonary fibrosis; HNSCC=head and neck squamous cell carcinoma; ca.=carcinoma.

for a summary).

Tanimoto *et al* (2000) had also studied OSCCs, as well as premalignant lesions for aberrant FHIT RT–PCR products. Evidence of aberrant transcripts was found in 53% of OSCCs and in two of seven premalignant lesions, one of which was from a patient who developed OSCC during follow-up. In a study of OSCCs associated with betel and/or tobacco use (Chang *et al*, 2002), 28% of samples exhibited FHIT promoter methylation, and 36% of cancers and 50% of premalignant lesions showed aberrant RT–PCR products, suggesting that FHIT aberration could be an early event in oral carcinogenesis. Reduced Fhit expression was observed in 41% of OSCCs. To determine the role of FHIT in tongue cancers, Fhit expression was studied by immunohistochemistry in sections from 41 stage II–IV squamous cell tongue carcinomas (Lee JI *et al*, 2001). Lost or decreased Fhit expression was demonstrated in 68% of tumours, especially in poorly differentiated areas. Patients whose tumours showed low or no Fhit had significantly shorter disease-free survival times than those with high Fhit-expressing tumours. Thus, loss of Fhit could be an independent prognostic indicator of clinical outcome for tongue cancer patients. Consistently with these findings, Rosin *et al* (2002) have shown that detection of allelic loss at the FHIT locus and/or 9p21 is a simple test for predicting a second oral malignancy at previously treated oral cancer sites.

Similarly, frequent LOH at 3p has been observed in head and neck squamous cell carcinoma (HNSCC). Mineta *et al* (2003) examined 57 HNSCCs by immunohistochemistry, Western blot and RT–PCR amplification for alterations in Fhit expression and looked for association with clinicopathologic features. Low Fhit expression was observed in 53% of cancers and correlated with Ki-67 expression, an indicator of aggressive proliferation.

Moving down to the oesophagus, FHIT LOH and Fhit expression were studied in precarcinomatous lesions and carcinoma. Primary tumours (76%) showed LOH encompassing FHIT and 70% were negative for Fhit protein. Tumours of patients who were heavy users of tobacco and alcohol showed significantly higher frequency of loss of Fhit expression in this study. Noncancerous squamous epithelia were mostly positive for Fhit, but five samples from heavy tobacco/alcohol users were Fhit negative. In addition, most carcinomas *in situ* (CIS), 50% of severe and moderate dysplasias and 33% of mild dysplasia were Fhit negative, suggesting that Fhit loss is an early event in ESC development (Mori *et al*, 2000).

Shimada *et al* evaluted the clinical impact of FHIT gene alterations in 149 ESCs by immunohistochemical analysis, and examined correlation with smoking history. Normal Fhit expression was observed in only 22% of cases, reduced expression in 45% and absence of Fhit in 33%. Fhit was also markedly reduced in muscle invasive tumours. This investigation did not find an association of Fhit loss with prognosis or smoking history. In a somewhat smaller study that included CIS and dysplasia in sections of 75 ESCs, Fhit protein was reduced, relative to adjacent normal mucosa, in 89% of invasive ESCs, 68% of CIS lesions and 43.5% of dysplastic lesions, so that Fhit loss was associated with progressive ‘increases in severity of histopathological changes’ (Kitamura *et al*, 2001).

We believe that these findings concerning oral and oesphageal cancers are very important. The treatment of these cancers can be disfiguring and debilitating and recurrence is common. These cancers are highly accessible for topical treatment using gene therapy approaches to prevention and may be candidates for future clinical trials, possibly using viral FHIT delivery.

The results of a number of recent reports of FHIT alterations in gastric cancer are also briefly summarised in Table 1. To determine if FHIT alterations are frequent in gastric carcinomas, Lee SH *et al* (2001) examined FHIT LOH, aberrant RT–PCR products and Fhit protein expression in 35 gastric adenocarcinomas. Aberrant transcripts were detected in 57% and Fhit protein reduction in 63%. Caselli *et al* (2001) studied preneoplastic lesions in histological samples of patients who developed gastric cancer within 2 years and did not observe reduction or loss of Fhit expression, whereas reduced or absent expression was observed in 61.5% of the cancers; complete Fhit loss was observed only in areas of low differentiation. Huiping *et al* observed absence or reduction of Fhit expression in 78% of gastric cancers and found an association between abnormal Fhit expression and positive node status. In summary, Fhit loss is frequent in gastric cancer, may not be an early event and its prognostic value has not yet been established.

## COLON CANCER

When the FHIT locus was first discovered, we noted frequent homozygous deletions in colon cancer cell lines and aberrant RT–PCR products in primary colorectal cancers (Ohta *et al*, 1996). Since then a number of investigators have examined the status of the FHIT gene and protein in colon cancer (see Table 1 for a summary). Hao *et al* (2000) examined aberrant crypt foci (ACF), adenomas, primary colorectal carcinomas (CRCs) and metastatic lesions for Fhit protein expression by immunohistochemistry. In all, 44% of carcinomas showed marked loss or absence of expression and the fraction of tumours with reduced expression increased with decreasing differentiation and in tumours with metastases (62 *vs* 38% in tumours without metastases); 12 out of 13 metastatic lesions showed reduced expression. Only a small fraction of ACFs and adenomas showed reduced Fhit, but reduced expression was strongly associated with degree of dysplasia. These authors suggested that Fhit plays a role in development and progression from the premalignant stage through metastasis.

Germline mutations in mismatch repair genes, usually MLH1 and MSH2, cause hereditary nonpolyposis colon cancer (HNPCC). We had been interested in a possible connection between defective mismatch repair and loss of Fhit expression because Fhit knockout mice develop sebaceous tumours of the skin (Fong *et al*, 2000), as do HNPCC carriers with Muir–Torre syndrome, involving sebaceous tumours of the skin combined with colon tumours. We also know that Msh2-deficient mouse cell lines show increased chromosome fragility (Turner *et al*, 2002), suggesting that intact mismatch repair systems are important for stability of fragile loci. Consistently with this idea, several reports of increased Fhit loss in mismatch repair-deficient colon cancers have appeared. Mori *et al* (2001), in an analysis of 62 CRC cases by immunohistochemistry for Fhit and Msh2 protein, found that Fhit loss correlated significantly with progression of carcinoma, as well as with lymph node metastasis, and loss of Msh2 correlated with loss of Fhit. Loss of Fhit occurred in 50% of sporadic CRCs and was more frequent in advanced cancers. It was concluded that mismatch repair protein may be important in maintaining the integrity of the common fragile locus within the FHIT gene. Similarly, Andachi *et al* (2002) reported that reduced Fhit expression is associated with mismatch repair deficiency in advanced colorectal carcinoma.

Interestingly, in a study of biopsies of periocular sebaceous gland carcinomas, of patients with Muir–Torre syndrome, Holbach *et al* (2002) determined that Fhit was detectable in the one sebaceous gland carcinoma with microsatellite instability but not in the five sebaceous carcinomas without microsatellite instability. These authors made the intriguing suggestion that loss of either Fhit or mismatch repair could contribute to the development of sebaceous gland carcinoma in MTS.

Finally, in a study employing computerised image analysis to quantitatively evaluate Fhit expression and apoptotic role in CRCs, Mady and Melhem (2002) concluded that absence or reduction of Fhit plays a role in the development of ∼23% of CRCs and was directly correlated with the occurrence of distant metastases and worse prognosis. Also, overexpression of Fhit was directly proportional to the apoptotic rate.

Thus, the results of each study were consistent with the conclusion that loss of Fhit protein expression, through damage to the FRA3B fragile locus, has an important role in the development of a significant fraction of colon cancers.

## CERVICAL CANCER

Cervical cancer was the first to be assessed for Fhit protein expression by immunohistochemistry and Fhit loss or reduction was observed in ∼70% of cervical cancers, a loss that correlated with the detection of aberrant FHIT RT–PCR amplification products. In a follow-up study that included preinvasive lesions, Connolly *et al* (2000) examined Fhit expression in 95 invasive cervical carcinomas, 33 high-grade squamous intraepithelial lesions (HSILs) associated with concurrent cancer, 38 HSILs without associated cancer and 24 low-grade squamous intra-epithelial (LSILs) lesions. Normal and LSIL samples showed moderate to strong cytoplasmic staining while Fhit staining was reduced or absent in 71% of invasive cancers, 52% of HSILs associated with invasive cancer and only 21% of HSILs without associated cancer. The conclusion was that loss of Fhit in HSILs could serve as a marker of high-grade preinvasive lesions that are likely to progress to invasive carcinoma.

In a study of 59 stage II–III tumours, absent or reduced Fhit protein was observed in 66% (Krivak *et al*, 2001) and multivariate analysis showed that reduced Fhit expression was a poor prognostic factor. The 3-year survival rate for patients with Fhit-positive tumours was 74 *vs* 37% for those with low Fhit-expressing tumours.

Guo *et al* (2001) analysed intratumoural heterogeneity of cervical cancers by studying 3p deletions and X-chromosome inactivation patterns in multiple microdissected samples from individual cancers. Allelic losses were frequently detected at 3p14.2 (FHIT), 3p21.3–21.2 and 3p24.2 markers and had occurred in CIN lesions synchronous with invasive lesions. Although the study involved normal and lesional DNAs from only 14 cervical cancers, the authors concluded that the ‘findings suggest essential roles of genes on these 3p loci, particularly the FHIT gene, in participating in clonal selection and early development of cervical cancer’.

Butler *et al* (2002) also studied CIN and microinvasive carcinoma (MICA) for expression of Fhit protein by immuno-histochemistry and looked for association of Fhit loss with clinical parameters, including high-risk HPV infection; 50% of CIN3 and 78% of MICA lesions showed reduction or absence of Fhit protein, while CIN1 lesions showed moderate to strong Fhit expression. A significant association was observed between loss of Fhit expression and HPV16 infection in the combined CIN and MICA lesions. A number of reports have looked for a correlation of loss of Fhit expression with presence of high-risk HPV genomes; some have found such a correlation and others have not (see Table 1 for a summary). Thus, this issue will need further, larger studies for resolution.

## LUNG CANCER

Owing to the connection between carcinogen exposure and Fhit loss, Fhit involvement in lung cancer was studied rather extensively early after discovery of the FHIT gene (reviewed in Huebner and Croce, 2002). In the last 3 years, there have been further reports on Fhit expression in lung cancer. Geradts *et al* (2000) aimed to correlate loss of Fhit expression with molecular genetic and clinical parameters in sections of 99 NSCLCs; 53% of tumours lacked Fhit staining, a lack that correlated with LOH at the FHIT locus. Fhit loss was as frequent as abnormalities of expression of p53, RB and p16 and occurred independently of most clinincal parameters and molecular abnormalities. Pavelic *et al* (2001) also examined the status of the FHIT gene in lung cancer and HNSCCs and compared it to expression of p21, frequency of apoptosis and proliferation. Most malignant lung and HNSCC lesions showed aberrant FHIT expression, reduced or absent p21 and increased cell proliferation. Fhit negativity tended to correlate with a worse prognosis (22.46 months median survival *vs* 36.04 months for Fhit-positive cases) and the trend was significant for HNSCCs (30.86 months median survival *vs* 64.04 months for Fhit-positive cases, *P*<0.05). Thus, aberrant Fhit might be a prognostic marker for lung cancer and HNSCC.

Garinis *et al* (2001) studied 67 NSCLCs and observed FHIT LOH in 64%, for both squamous and adenocarcinomas. Allelic imbalance at FHIT was 71% in stage I cancers, showing early involvement. There was no association with kinetic parameters or ploidy of tumours, but concurrent loss of Fhit and overexpression of p53 was observed in 39%. Authors suggested that FHIT allele losses could be the outcome of tobacco-induced mutagenesis. Pylkkanen *et al* (2002) aimed to determine whether absent or reduced Fhit or FHIT allele loss was associated with exposure to lung carcinogens. Reduced Fhit expression was observed in 62% of cases and was common in asbestos-exposed (67%) and nonexposed cases (59%). FHIT LOH was increased in advanced disease and in poorly differentiated tumours, supporting the significance of FHIT inactivation in lung cancer development.

## BREAST CANCER

The cytogenetics of breast cancer has recently been reviewed for 322 karyotypically abnormal samples from 256 patients (Teixeira *et al*, 2002). ‘Interstitial deletions of the short arm of chromosome 3, with 3p14 as a minimally common deleted region, were described as a recurrent change in breast carcinoma by Pandis *et al*…. and have turned out to be the single most common structural rearrangement’…. Also, it occurs usually as the sole clonal abnormality and in benign proliferative disease of the breast. Molecular studies have confirmed loss of 3p in breast cancers, with loss at 3p13–14 having been reported in more than 40% of breast carcinomas (Teixeira *et al*, 2002). ‘A candidate tumour suppressor gene in this region is FHIT and some studies have shown that this gene is involved in breast cancer…’.

Thus, although breast cancer cytogenetics is notoriously complex, loss of 3p material, including the FHIT region, is an early event in a fraction of breast cancers, and homozygous loss of at least a portion of the FHIT gene was observed in a case of bilaterial breast cancer, as were aberrant FHIT RT–PCR products and reduced Fhit expression in several series of sporadic breast cancers (reviewed in Huebner *et al*, 1998).

More recently, Kollias *et al* (2000) tested for concordant changes in left and right breast cancers of young women with bilateral cancer. Microsatellite markers were used to test for LOH at candidate genes TP53, BRCA1, BRCA2, ATM and FHIT. Four cases showed concordant loss of BRCA1 alleles in left and right cancers, four for BRCA2, seven for ATM and four cases for FHIT, suggesting possible roles for these tumour suppressor genes.

Several early studies of alterations at the FHIT locus in breast cancer reported reduced expression of Fhit in ∼40–60% of mammary carcinomas and an elevated frequency of loss in BRCA2-linked breast carcinomas (Huebner and Croce, 2001). The higher frequency of alteration of FRA3B and reduced expression of Fhit in BRCA2-linked cancers was consistent with the idea that loss of BRCA2 function affects stability of the FRA3B/FHIT locus. This theme was pursued in a study of BRCA1-linked breast cancers (Turner *et al*, 2002); breast tumours with deleterious BRCA1 mutations were analysed for Fhit expression by immunohistochemistry. Loss of Fhit expression was significantly more frequent in the BRCA1 cancers compared with sporadic breast tumours (9% Fhit positive *vs* 68% Fhit positive), suggesting that the BRCA1 pathway is also important in protecting the FRA3B/FHIT locus from damage. To further investigate the relation between repair gene deficiencies and induction of fragile sites *in vitro*, the frequency of aphidicolin-induced chromosome gaps and breaks was analysed in PMS2-, BRCA1-, MSH2-, MLH1-, FHIT- and TP53-deficient cell lines. Each repair-deficient cell line showed elevated chromosome gaps and breaks, consistent with the proposal that proteins involved in repair are important in maintaining the integrity of common fragile regions (Turner *et al*, 2002). Correspondingly, genes at common fragile sites may sustain elevated levels of DNA damage in cells with deficient DNA repair proteins such as those mutated in several familial cancer syndromes.

Ingvarsson *et al* (2001) have examined the relation between FHIT LOH and breast tumour progression. FHIT gene markers were typed in 239 breast tumours and paired normal tissue, and results assessed relative to clinicopathologic factors and LOH at other regions. This study found that FHIT LOH was associated with oestrogen and progesterone negativity, high S-phase fraction, reduced patient survival and LOH at other chromosome regions. Perhaps most interestingly, FHIT LOH resulted in a 60% increased relative risk of death. The overall conclusion was that FHIT LOH results in growth advantage of breast tumour cells, is associated with an unstable genome and may be of prognostic value.

Similarly, Yang *et al* (2001), in a study of more than 160 breast cancers occurring in Asian women, found reduction of Fhit expression in 42%, and compared expression levels with clinicopathological profiles and expression of other biological markers. Reduced Fhit expression was significantly associated with histological grade, high tumour proliferation, negative ER status and p53 overexpression. Patients with tumours with loss of Fhit expression tended to have poor survival. Results of these recent breast cancer studies are summarised in Table 1.

## CONCLUSION AND PERSPECTIVE

The data summarised indicate that the tumour suppressor gene, FHIT, is altered in many human tumours, particularly in those caused by environmental carcinogens, such as those present in tobacco smoke. In many of these tumours, particularly in those induced by tobacco or other environmental carcinogens, alterations of FHIT occur very early during the multistep process of carcinogenesis.

We have also shown that Fhit-negative cancer cells are very sensitive to the expression of FHIT; for example, infection with FHIT recombinant viruses (Huebner and Croce, 2001) can cause regression and prevention of tumours in experimental animals. Thus, it is logical to predict the development of a gene therapy approach for the treatment and prevention of Fhit-negative human cancers.

## References

[bib1] Andachi H, Yashima K, Koda M, Kawaguchi K, Kitamura A, Hosoda A, Kishimoto Y, Shiota G, Ito H, Makino M, Kaibara N, Kawasaki H, Murawaki Y (2002) Reduced Fhit expressions associated with mismatch repair deficiency in human advanced colorectal carcinoma. Br J Cancer 87: 441–445 12177781 10.1038/sj.bjc.6600501 PMC2376126

[bib2] Butler D, Collins C, Mabruk M, Leader MB, Kay EW (2002) Loss of Fhit expression as a potential marker of malignant progression in preinvasive squamous cervical cancer. Gynecologic Oncol 86: 144–149 10.1006/gyno.2002.6712 12144820

[bib3] Caselli M, Marchisio M, Gaudio M, Saragoni L, Lanza G, Alvisi V, Bertagnolo V, Concu M, Capitani S, Caramelli E (2001) Fhit protein expression in human gastric cancer and related precancerous lesions. Oncol Rep 8: 1233–1237 11605039 10.3892/or.8.6.1233

[bib4] Chang KW, Kao SY, Tzeng RJ, Liu CJ, Cheng AJ, Yang SC, Wong YK, Lin SC (2002) Multiple molecular alterations of FHIT in betel-associated oral carcinoma. J Pathol 196: 300–306 11857493 10.1002/path.1047

[bib5] Connolly DC, Greenspan DL, Wu R, Ren X, Dunn RL, Shah KV, Jones RW, Bosch FX, Munoz N, Cho KR (2000) Loss of Fhit expression in invasive cervical carcinomas and intraepithelial lesions associated with invasive disease. Clin Cancer Res 6: 3505–3510 10999736

[bib6] Dumon KR, Ishii H, Fong LYY, Zanesi N, Fidanza V, Vecchione, A, Baffa R, Trapasso F, During MJ, Huebner K, Croce CM (2001) FHIT gene therapy prevents tumour development in Fhit-deficient mice. Proc Natl Acad Sci USA 98: 3346–3351 11248081 10.1073/pnas.061020098 PMC30656

[bib7] Fong LYY, Fidanza V, Zanesi N, Lock L, Siracusa L, Mancini R, Siprashvilli Z, Ottey M, Martin SE, Druck T, McCue PA, Croce CM, Huebner K (2000) Muir–Torre-like syndrome in Fhit deficient mice. Proc Natl Acad Sci USA 97: 4742–4747 10758156 10.1073/pnas.080063497 PMC18303

[bib8] Garinis GA, Gorgouli VG, Mariatos G, Zacharatos P, Kotsinas A, Liloglou T, Foukas P, Kanavaros P, Kastrinakis NG, Vassilakopoulas T, Vogiatzi T, Field JK, Kittas C (2001) Association of allelic loss at the FHIT locus and p53 alterations with tumour kinetics and chromosomal instability in non-small cell lung carcinomas (NSCLCs). J Pathol 193: 55–65 11169516 10.1002/1096-9896(2000)9999:9999<::AID-PATH731>3.0.CO;2-#

[bib9] Geradts J, Fong KM, Zimmerman PV, Minna JD (2000) Loss of Fhit expression in non-small-cell long cancer: correlation with molecular genetic abnormalities and clinicopathological features. Br J Cancer 82: 1191–1197 10735505 10.1054/bjoc.1999.1062 PMC2363352

[bib10] Guo Z, Wu F, Asplun A, Hu X, Mazurenko N, Kisseljov F, Ponten J, Wilander E (2001) Analysis of intratumorial heterogeneity of chromosome 3p deletions and genetic evidence of polyclonal origin of cervical squamous carcinoma. Mol Pathol 14: 54–61 10.1038/modpathol.3880256 11235906

[bib11] Hao XP, Willis JE, Pretlow TG, Rao JS, MacLennan GT, Talbot IC, Pretlow TP (2000) Loss of fragile histidine triad expression in colorectal carcinomas and premalignant lesions. Cancer Res 60: 18–21 10646844

[bib12] Helland A, Kraggerud SM, Kristensen GB, Holm R, Abeler VM, Huebner K, Borresen-Dale AL, Lothe RA (2000) Primary cervical carcinomas show 2 common regions of deletion at 3p, 1 within the FHIT gene: evaluation of allelic imbalance at FHIT, RB1 and TP53 in relation to survival. Int J Cancer 88: 217–222 11004671 10.1002/1097-0215(20001015)88:2<217::aid-ijc11>3.0.co;2-i

[bib13] Herzog CR, Crist KA, Sabourin CL, Kelloff GJ, Boone CW, Stoner GD, You M (2001) Chromosome 3p tumour-suppressor gene alterations in cervical carcinoma. Mol Carcinogen 30: 159–168 10.1002/mc.1024 11301476

[bib14] Holbach LM, von Moller A, Decker C, Junemann AG, Rummelt-Hofmann C, Ballhausen WG (2002) Loss of fragile histidine triad (FHIT) expression and microsatellite instability in periocular sebaceous gland carcinoma in patients with Muir–Torre syndrome. Am J Ophthalmol 134: 147–148 12095833 10.1016/s0002-9394(02)01434-4

[bib15] Huebner K, Croce CM (2002) FRA3B and other common fragile sites: The weakest Links. Nat Rev Cancer 1: 214–221 10.1038/35106058 11902576

[bib16] Huebner K, Garrison PN, Barnes LD, Croce CM (1998) The role of the *FRA3B*/*FHIT* locus in cancer. Ann Rev Genet 32: 7–31 9928473 10.1146/annurev.genet.32.1.7

[bib17] Huiping C, Kristjansdottir S, Bergthorsson JT, Jonasson JG, Magnusson J, Egilsson V, Ingvarsson S (2002) High frequency of LOH, MSI and abnormal expression of FHIT in gastric cancer. Eur J Cancer 38: 728–735 11916557 10.1016/s0959-8049(01)00432-4

[bib18] Ingvarsson S, Sigbjornsdottir BI, Huiping C, Jonasson JG, Agnarsson BA (2001) Alterations of the FHIT gene in breast cancer: association with tumour progression and patient survival. Cancer Detect Prev 25: 318–324 11425271

[bib19] Kitamura A, Yashima K, Okamoto E, Andachi H, Hosoda A, Kishimoto Y, Shiota G, Ito H, Kaibara N, Kawasaki H (2001) Reduced Fhit expression occurs in the early stage of esophageal tumorigenesis: no correlation with p53 expression and apoptosis. Oncology 61: 205–211 11574776 10.1159/000055376

[bib20] Kollias J, Man S, Marafie M, Carpenter K, Pinder S, Ellis IO, Blamey RW, Cross G, Brook JD (2000) Loss of heterozygosity in bilateral breast cancer. Breast Cancer Res Treat 64: 241–251 11200774 10.1023/a:1026575619155

[bib21] Krivak TC, McBroom JW, Seidman J, Venzon D, Crothers B, MacKoul PJ, Rose GS, Carlson JW, Birrer MJ (2001) Abnormal fragile histidine triad (FHIT) expression in advanced cervical carcinoma: a poor prognostic factor. Cancer Res 61: 438–485 11389064

[bib22] Lee JI, Soria JC, Hassan K, Liu D, Tang X, El-Naggar A, Hong WK, Mao L (2001) Loss of Fhit expression is a predictor of poor outcome in tongue cancer. Cancer Res 61: 837–841 11221865

[bib23] Lee SH, Kim WH, Kim HK, Woo KM, Nam HS, Kim HS, Kim JG, Cho MH (2001) Altered expression of the fragile histidine triad gene in primary gastric adenocarcinomas. Biochem Biophys Res Commun 284: 850–855 11396980 10.1006/bbrc.2001.5038

[bib24] Luceri C, Guglielmi F, De Filippo C, Caderni G, Mini E, Biggeri A, Napoli C, Tonelli F, Cianchi F, Dolara P (2000) Clinicopathologic features and FHIT gene expression in sporadic colorectal adenocarcinomas. Scand J Gastroenterol 35: 637–641 10912665 10.1080/003655200750023615

[bib25] Mady HH, Melhem MF (2002) FHIT protein expression and its relation to apoptosis, tumour histologic grade and prognosis in colorectal adenocarcinoma: an immunohistochemical and image analysis study. Clin Exp Metast 19: 351–358 10.1023/a:1015594702522 12090476

[bib26] Menin C, Santacatterina M, Zambon A, Montagna M, Parenti A, Ruol A, D'Andrea E (2000) Anomalous transcripts and allelic deletions of the FHIT gene in human esophageal cancer. Cancer Genet Cytogenet 119: 56–61 10812172 10.1016/s0165-4608(99)00216-2

[bib27] Mineta H, Miura K, Takebayashi S, Misawa K, Ueda Y, Suzuki I, Ito M, Wennerberg J (2003) Low expression of fragile histidine triad gene correlates with high proliferation in head and neck squamous cell carcinoma. Oral Oncol 39: 56–63 12457722 10.1016/s1368-8375(02)00022-2

[bib28] Mori M, Mimori K, Masuda T, Yoshinaga K, Yamashita K, Matsuyama A, Inoue H (2001) Absence of Msh2 protein expression is associated with alteration in the FHIT locus and Fhit protein expression in colorectal carcinoma. Cancer Res 61: 7379–7382 11606365

[bib29] Mori M, Mimori K, Shiraishi T, Alder H, Inoue H, Tanaka Y, Sugimachi K, Huebner K, Croce CM (2000) Altered expression of Fhit in carcinoma and precarcinomatous lesions of the esophagus. Cancer Res 60: 1177–1182 10728669

[bib31] Morikawa H, Nakagawa Y, Hashimoto K, Niki M, Egashira Y, Hirata I, Katsu K, Akao Y (2000) Frequent altered expression of fragile histidine triad protein in human colorectal adenomas. Biochem Biophys Res Commun 278: 205–210 11071873 10.1006/bbrc.2000.3771

[bib132] Ohta M, Inoue H, Cotticelli MG, Kastury K, Baffa R, Palazzo J, Siprashvili Z, Mori M, McCue P, Druck T, Croce CM, Huebner K (1996) The FHIT gene spanning the chromosome 3p14.2 fragile site and renal carcinoma-associated t(3;8) breakpoint is abnormal in digestive tract cancers. Cell 84: 587–597 8598045 10.1016/s0092-8674(00)81034-x

[bib32] Pavelic K, Krizanac S, Cacev T, Hadzija MP, Radosevic S, Crnic I, Levanat S, Kapitanovic S (2001) Aberration of FHIT gene is associated with increased tumour proliferation and decreased apoptosis–clinical evidence in lung and head and neck carcinomas. Mol Med 7: 442–453 11683369 PMC1950052

[bib33] Pylkkanen L, Wolff H, Stjernvall T, Tuominen P, Sioris T, Karjalainen A, Anttila S, Husgafvel-Pursiainen K (2002) Reduced Fhit protein expression and loss of heterozygosity at FHIT gene in tumours from smoking and asbestos-exposed lung cancer patients. Int J Oncology 20: 285–290 11788890

[bib34] Rosin MP, Lam WL, Poh C, Le ND, Li RJ, Zeng T, Priddy R, Zhang L (2002) 3p14 and 9p21 loss is a simple tool for predicting second oral malignancy at previously treated oral cancer sites. Cancer Res 62: 6447–6450 12438233

[bib35] Shimada Y, Sato F, Watanabe G, Yamasaki S, Kato M, Maeda M, Imamura M. (2000) Loss of fragile histidine triad gene expression is associated with progression of esophageal squamous cell carcinoma, but not with the patient's prognosis and smoking history. Cancer 89: 5–11 10896994

[bib36] Tanimoto K, Hayashi S, Tsuchiya E, Tokuchi Y, Kobayashi Y, Yoshiga K, Okui T, Kobayashi M, Ichikawa T (2000) Abnormalities of the FHIT gene in human oral carcinogenesis. Br J Cancer 82: 838–843 10732756 10.1054/bjoc.1999.1009 PMC2374395

[bib37] Teixeira MR, Pandis N, Heim S (2002) Cytogenetic clues to breast carcinogenesis. Genes Chrom Cancer 33: 1–16 11746982 10.1002/gcc.1206

[bib38] Terry G, Ho L, Londesborough P, Cuzick J (2002) Abnormal FHIT expression profiles in cervical intraepithelial neoplastic (CIN) lesions. Br J Cancer 86: 376–381 11875703 10.1038/sj.bjc.6600077 PMC2375220

[bib39] Turner B, Ottey M, Potoczek M, Hauck WW, Pequignot E, Keck-Waggoner C, Zimonjic DB, Sevignani C, Aldaz M, McCue P, Palazzo J, Huebner K, Popescu NC (2002) The *FHIT*/*FRA3B* locus and repair deficient cancers. Cancer Res 62: 4054–4060 12124341

[bib40] van Heerden WFP, Swart TJP, Robson B, Smith T-L, Engelbrecht S, van Heerden MB, van Rensburg EJ, Huebner K (2001) *FHIT* RNA and protein expression in oral squamous cell carcinomas. Anticancer Res 21: 2425–2428 11724302

[bib41] Yang Q, Yoshimura G, Suzuma T, Tamaki T, Umemura T, Nakamura M, Nakamura Y, Wang X, Mori I, Sakurai T, Kakudo K (2001) Clinicopathological significance of fragile histidine triad transcription and protein expression in breast carcinoma. Clin Cancer Res 7: 3869–3873 11751477

